# High-density lipoprotein cholesterol: how studying the ‘good cholesterol’ could improve cardiovascular health

**DOI:** 10.1098/rsob.240372

**Published:** 2025-02-19

**Authors:** Lucy Diaz, Ewa Bielczyk-Maczynska

**Affiliations:** ^1^ The Hormel Institute, University of Minnesota, Austin, MN, USA; ^2^ The Institute for Diabetes, Obesity, and Metabolism, University of Minnesota, Minneapolis, MN, USA; ^3^ Lillehei Heart Institute, University of Minnesota, Minneapolis, MN, USA

**Keywords:** lipoprotein, receptor, high-density lipoprotein, cholesterol uptake

## Abstract

High cholesterol levels are associated with an increased risk of cardiovascular disease, specifically atherosclerosis, a leading cause of death worldwide. Atherosclerosis occurs when cholesterol and fat build up in plaques along blood vessel walls, restricting blood flow and preventing nutrients and oxygen from diffusing in and out of the bloodstream. High-density lipoprotein cholesterol (HDL) particles prevent the build-up of such plaques, removing excess cholesterol from the peripheral tissues and delivering it to the liver, where it can be removed from the body. This pathway is known as reverse cholesterol transport (RCT). Because HDL plays a key role in preventing plaque buildup, understanding how this molecule and RCT function in the body could help us develop much-needed new atherosclerosis therapies and prevention strategies. However, HDL metabolism is complex, and research on HDL has been less favoured than research investigating a much better-understood molecule, low-density lipoprotein cholesterol, as a treatment target. More specifically, the receptors involved in the process of taking up HDL within the liver and their relationships to one another, along with the mechanism of whole, or holoparticle uptake of HDL remain to be clarified. In this review, we discuss several outstanding mysteries in HDL metabolism, consider why previous clinical trials to improve cardiovascular health by modulating HDL levels have been unsuccessful and argue that understanding HDL metabolism is essential for crafting interventions to reduce cardiovascular disease risk.

**Figure:**
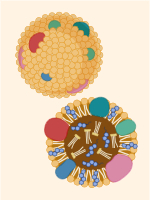

## 1. High-density lipoprotein cholesterol: a neglected molecule in the fight against atherosclerosis

The main types of cholesterol present in the blood are low-density lipoprotein cholesterol (LDL-C) and high-density lipoprotein cholesterol (HDL-C). LDL-C is sometimes referred to as the ‘bad cholesterol’, because the blood LDL-C concentrations are positively correlated with risk of cardiovascular disease and events [1]. Due to its strong correlation with cardiovascular disease, LDL-C has been extensively studied. HDL-C, sometimes known as the ‘good cholesterol’, has had less success as a clinical target, likely due to its more complex role. HDL-C plays a key part in reverse cholesterol transport (RCT), the process by which excess cholesterol is removed from the bloodstream, preventing the buildup of arterial plaques. Low plasma HDL levels are associated with impaired RCT, but increased HDL levels alone do not appear to confer significant health benefits [2].

Removing excess cholesterol and fat from the bloodstream is essential for maintaining cardiovascular health. If excess cholesterol is not removed efficiently, cholesterol plaques form along artery walls, making arteries stiff and clogged and leading to atherosclerosis [3–5]. Atherosclerosis, in turn, can lead to heart attack, stroke and heart or kidney failure [3,5]. It is also linked to obesity and diabetes, both of which are on the rise globally [6–8]. Between 1990 and 2019, the number of new cases of cardiovascular disease rose by 77%, with ageing and urbanization trends drastically increasing the number of cases [9]. Statins are the most commonly used treatment for atherosclerosis and function via decreasing LDL-C [10]. However, levels of LDL-C are not the sole contributor to development of atherosclerosis, and are intrinsically linked to levels of other metabolites, such as HDL-C. The disease burden of atherosclerosis is likely to continue to rise across the globe, and more effective treatments are necessary to effectively address the different causes of the disease. Statins are not a catch-all cure, and lowering LDL-C alone is likely not the most effective strategy for the treatment and prevention of atherosclerosis and cardiovascular disease.

Indeed, the relationship between HDL, RCT and cardiovascular disease is complex and poorly understood; we have identified only some of the molecular pathways and receptors involved in HDL metabolism [11,12]. Whereas LDL has one main receptor, the low-density lipoprotein receptor (LDLR), HDL-C can be taken up by multiple receptors, all of which are capable of binding to other particles within the blood, including LDL [13,14]. To add to the complexity of studying HDL, HDL and LDL levels in the blood are linked directly via the cholesteryl ester transfer protein (CETP) [15–17]. This can make it difficult to separate the effects of HDL-C or LDL-C alone [18]. Nevertheless, untangling the complicated molecular pathways that involve HDL-C and underlie RCT could potentially help us identify new druggable targets for atherosclerosis and related diseases and increase the efficiency of current LDL-C targeting treatments [19–21]. Here, we discuss several outstanding mysteries in HDL-C metabolism and suggest future steps for solving them. We argue that discovering more about the complex pathways involving HDL-C in the body and their influence on maintaining healthy cholesterol levels will pay great dividends by allowing us to develop novel interventions to address the early progression of atherosclerosis.

## 2. Clinical promise of manipulating high-density lipoprotein cholesterol levels for reducing atherosclerosis

Currently, statins are the most commonly used treatment for high cholesterol levels, but solely decrease LDL-C cholesterol and triglycerides [10], leaving open possibilities for the modulation of HDL-C for cardiovascular benefits. Due to the connection between low HDL-C levels and increased risk of cardiovascular disease [2], it has been hypothesized that increasing plasma HDL-C levels might lower the risk of atherosclerosis. In clinical trials, niacins and fibrates have been successful at increasing HDL-C [22,23]. However, the treatments frequently require high dosages, have side effects and are not suited for treating the lipid profile of a large percentage of patients [24–28]. This has caused the research community’s attention to shift to stimulating and increasing RCT capacity instead. Two therapeutic approaches have emerged from this line of thinking. The first approach is inhibiting *CETP* to prevent cholesteryl ester (CE) transfer from HDL to LDL, thus decreasing LDL-C levels, increasing plasma HDL-C and boosting cholesterol efflux rates. The second approach is increasing levels of ApoAI, the primary lipoprotein component of HDL whose levels are tied to the generation of new HDL particles [29].

Many different CETP inhibitors have been trialled in combination with statins in hopes of reducing the risk of cardiovascular events. CETP inhibition initially drew interest due to the elevated HDL-C levels and decreased LDL-C levels associated with CETP loss-of-function variants in humans, the beneficial effects of which have also been confirmed in mice and rabbits [30–32]. However, three different clinical trials of CETP inhibitors, torcetrapib [33,34], dalcetrapib [35] and evacetrapib, have been terminated [36,37]. Torcetrapib was terminated due to adverse off-targeting effects that have been remedied in subsequent CETP inhibitors, which have been terminated due to lack of efficacy [19]. Anacetrapib [38], another CETP inhibitor, significantly reduced the risk of major coronary events among patients with atherosclerotic vascular disease receiving intensive statin therapy in the phase 3 REVEAL trial [39]. However, Merck decided not to apply for regulatory approval of the drug because experts believed that the modest benefit observed in the trial was consistent with its effect on lowering LDL-C levels, rather than reflecting a novel HDL-C based mechanism [40]. Another remaining CETP inhibitor, obicetrapib, was tested for the same purpose in the phase 3 BROADWAY trial [41]. Results of this trial have yet to be published. However, in a phase 2 clinical trial, obicetrapib combined with the cholesterol-lowering medication ezetimibe and statin treatment significantly lowered atherogenic lipid and lipoprotein levels [41]. There is hope that the BROADWAY trial will replicate the combined improvement in HDL-C and other metabolic markers, and these improvements in proximal endpoints will translate to a reduced risk of cardiovascular events. Further results of the phase 3 BROADWAY trial are likely to guide future studies of lipoprotein metabolism, with the aim of targeting multiple types of cholesterol to improve cardiovascular outcomes.

Therapies that target ApoAI, the primary lipoprotein component of HDL-C that is responsible for binding to HDL-C receptors, are also being explored [42]. Increasing the quantity of available ApoAI for HDL synthesis and ApoAI-mediated cholesterol efflux could increase RCT rates and decrease blood vessel plaques and the risk of atherosclerosis. In cell culture and monkey models, the drug apabetalone increases ApoAI protein levels, increasing cholesterol efflux capacity and decreasing atherosclerotic plaque formation [43,44]. Unfortunately, the results in humans have been less promising [45]. In a phase 2 clinical trial conducted on patients with recent acute coronary syndrome, type 2 diabetes and low HDL-C levels who were receiving standard care, apabetalone increased HDL-C levels relative to placebo but did not significantly decrease the risk of cardiovascular events [46]. As expected, apabetalone had no influence on LDL-C levels. Beyond apabetalone, ApoAI overexpression was studied long-term in a mouse model, resulting in a sixfold reduction in the formation of atherosclerotic plaques, showing significant promise for the reduction of atherosclerosis via ApoAI increase [47]. Injection of ApoAI into mice [48] and rabbits [49] has also been documented, indicating that increasing the level of available ApoAI may be much simpler than other lipid-altering mechanisms, which shows promise as the disease burden of atherosclerosis continues to rise around the world [8]. Most recently, the international phase 3 clinical trial known as AEGIS-II concluded in late 2024, involving the infusion of human-purified ApoAI into patients who had previously experienced a heart attack, had multivessel coronary artery disease and/or increased cardiovascular risk factors [50,51]. The treated patient group showed numerically lower rates of cardiovascular death, heart attack and stroke compared to the placebo group at 90 days, and significantly lower rates at 180 and 365 days [50,51]. The difference in rates of cardiovascular death and non-type 2 heart attacks, the events most likely to be impacted by changes in ApoAI, were significant at all three time points, and showed increased variance from the placebo as more time passed [50].

To date, inhibiting CETP and targeting ApoAI to modulate HDL metabolism have been largely unsuccessful at minimizing cardiovascular event risk, though the recent results from the AEGIS-II trial show promise. However, these agents may still prove useful if combined with other novel agents that target other points in HDL-C metabolism, such as uptake by cells in the liver. Our lack of knowledge regarding the molecular basis of HDL-C’s role in cholesterol efflux has confined us to agents that target points upstream of receptor binding, where they impact a multitude of biological pathways likely to have feedback mechanisms that prevent these therapies from being truly effective. Further investigating the downstream effects of HDL-C binding to its various receptors and identifying the physiological factors that cause binding rates to shift could point researchers towards specific targets that can be combined with therapies that increase HDL-C levels. Human genetic variants that modulate the expression of SR-BI, the primary HDL receptor, have a bigger impact on cardiovascular disease than variants that alter HDL-C levels alone [52,53]. This suggests that modulating the activity of HDL-C receptors and other yet-to-be determined downstream players may have a considerable impact on relieving atherosclerotic burden, especially in combination with therapies that raise HDL-C levels. In short, due to the complexity of HDL metabolism, which includes many intertwined signalling pathways, it seems unlikely that therapeutically targeting one point in this process will yield significant results. Combining treatments that target multiple points in HDL and/or both LDL and HDL metabolism simultaneously may prove a more promising approach for reducing atherosclerosis risk, but to do so, we need to dig deeper into HDL metabolism.

## 3. An enduring mystery: how high-density lipoprotein cholesterol particles are taken up by liver cells to remove cholesterol from the body

Understanding how HDL-C is taken up by liver cells, so that cholesterol can be removed from the bloodstream, is critical for understanding HDL metabolism. However, many aspects of HDL-C uptake remain a mystery. HDL-C shepherds excess cholesterol from the bloodstream to the liver, where it is taken up by liver cells in one of two ways. In selective CE uptake, CEs and triglycerides within the centre of an HDL particle are extracted and delivered to liver cells, where they are converted to bile acids and excreted from the body. The HDL particle is then released from the liver cell back into the bloodstream, where it can continue to capture excess cholesterol and repeat this process. In holoparticle uptake, the entire HDL particle, including both its lipoprotein casing and its CE and triglyceride contents, is removed from the bloodstream, and enters liver or kidney cells. Here, we focus primarily on holoparticle uptake by hepatocytes because of the lack of knowledge surrounding the process in the liver. Unlike selective CE uptake rates, holoparticle uptake rates directly impact the level of HDL circulating in the bloodstream, where they collect cholesterol and prevent plaque formation.

**Figure 1.**
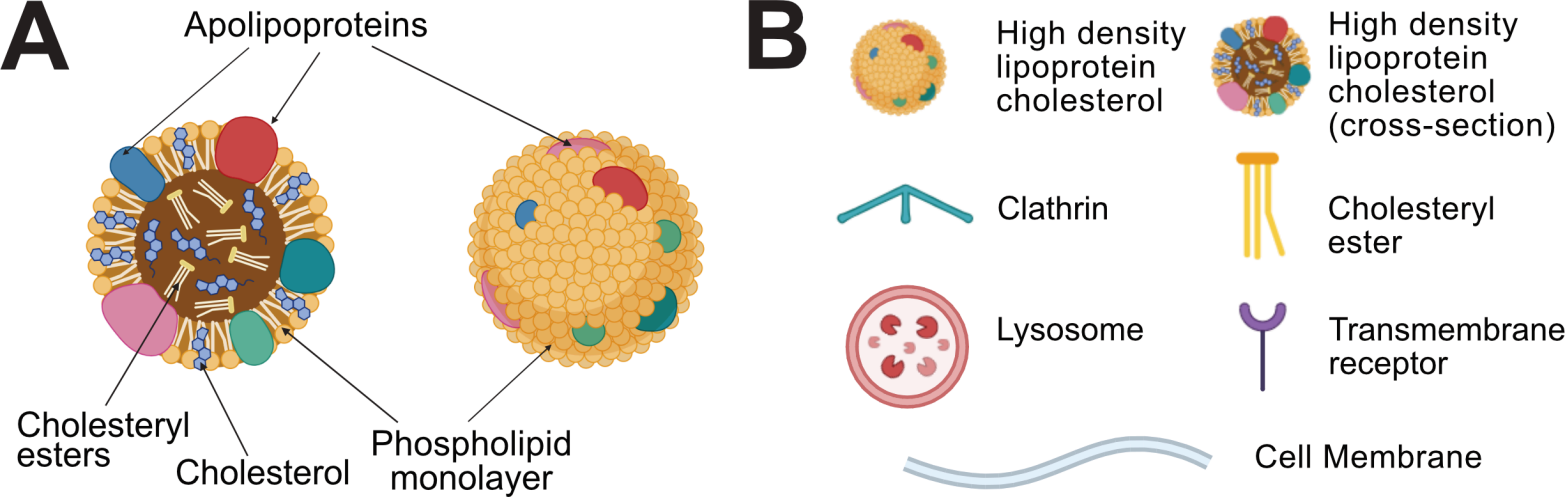
HDL-C and lipoprotein metabolism players. (A) Identification of the components of an HDL-C particle. (B) Key for later schematics of HDL-C uptake.

In both modes of HDL-C uptake, the HDL particle’s apolipoprotein exterior binds to receptors on the surface of liver cells (figure 1). The dominant component of the apolipoprotein exterior is typically ApoAI, which interacts with the scavenger receptor class B type 1 (SR-B1), the primary HDL-C receptor [54]. ApoE, a minor lipoprotein component of HDL-C, permits the binding of HDL-C to additional receptors [13,14,55]. The contribution of ApoE binding to HDL-C uptake will not be discussed in this review. However, as discussed below, the identity of all of the receptors that play a key role in HDL-C uptake remains unknown, as do many aspects of the processes that take place after HDL-C binds to the receptors.

## 4. Modulating high-density lipoprotein holoparticle uptake rates in the liver to combat atherosclerosis

As mentioned, HDL holoparticle uptake by the liver cells removes HDL particles from the bloodstream, preventing them from participating in RCT and potentially increasing cardiovascular disease risk. For this reason, understanding how HDL holoparticle uptake works is a subject of interest for researchers looking at HDL-C as a potential target for atherosclerosis treatment.

**Figure 2.**
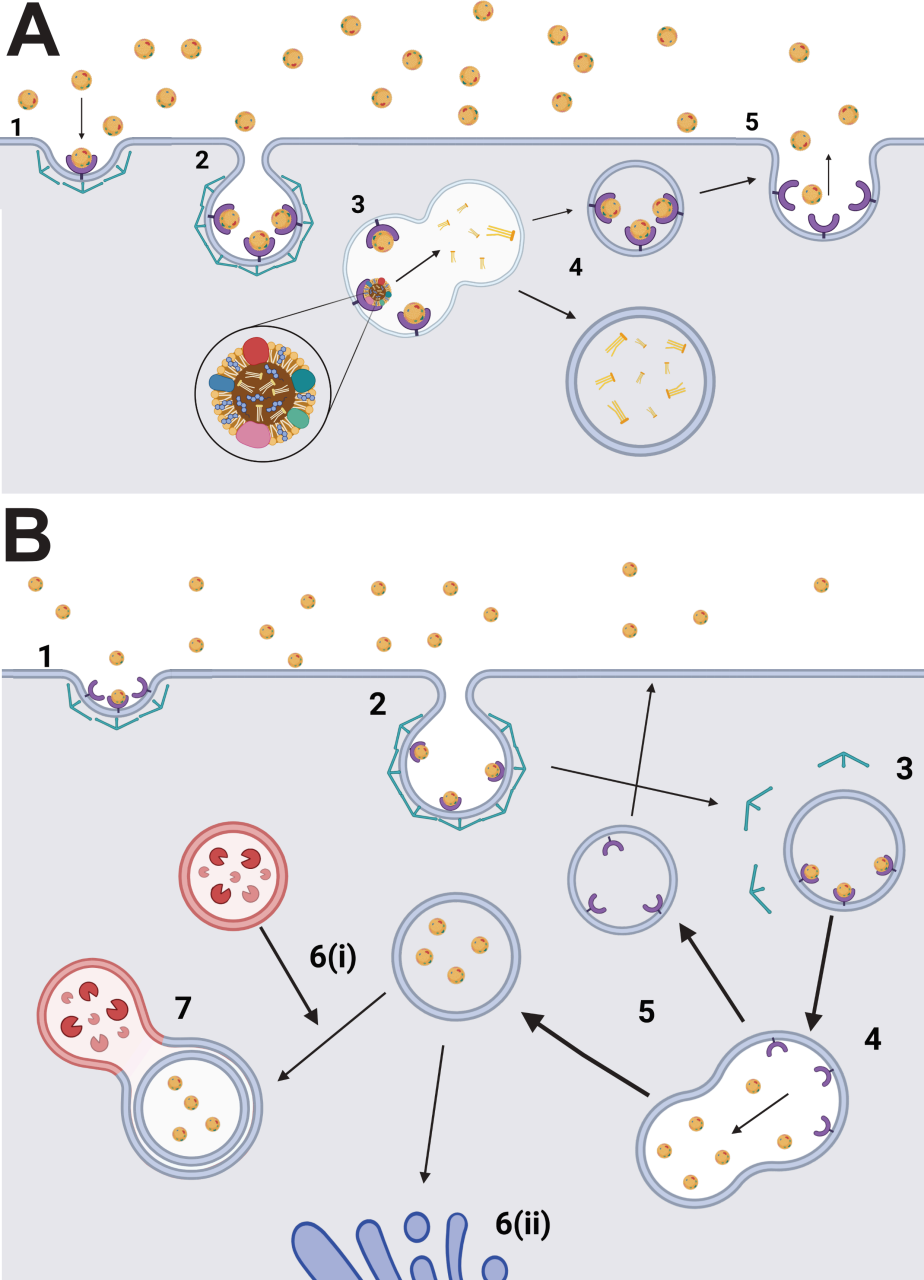
HDL-C receptor-mediated retroendocytosis versus holoparticle uptake. (A) (1 and 2) HDL-C binds to a transmembrane receptor and is taken into the cell via a clathrin-coated vesicle. (3) Cholesteryl esters are extracted from the HDL-C particles. (4) The receptors and cholesteryl esters are separated. (5) HDL-C particles and receptors are recycled back to the cell surface, where whole HDL-C particles are released back into the plasma. (B) (1 and 2) HDL-C particles are taken into the cell from the plasma via a transmembrane receptor into a clathrin-coated vesicle. (3) The vesicle is enveloped by the cell and clathrin disassociates, returning to the membrane. (4 and 5) HDL-C disassociates from the receptor, and the receptor is recycled back to the cell surface. (6(i) and 7) The HDL-C containing vesicle combines with a lysosome. (6(ii)) The HDL-C containing vesicle is remanded to the Golgi apparatus for processing.

HDL holoparticle uptake occurs in the liver [56], but the specific molecular pathway(s) responsible remain elusive. Holoparticle uptake is considered the entry of the entire HDL particle into the cell, including its lipoprotein casing and its CE and triglyceride contents (figure 2). It remains unclear whether holoparticle uptake can occur in conjunction with selective CE uptake or if it is an entirely separate process that can be conducted by some HDL-C receptors but not others [57]. As mentioned previously, the level of HDL-C in the bloodstream directly impacts its capability to function in RCT. Decreasing holoparticle uptake to increase levels of HDL-C in the blood could increase its atheroprotective effects, making HDL-C holoparticle uptake a subject of interest for those looking at HDL-C as a potential treatment target. However, sustained circulation of HDL holoparticles may alter their size and apolipoprotein composition, impacting cholesterol efflux, selective CE uptake capacity and ability to bind HDL-C receptors [58–60]. Therefore, a lack of HDL holoparticle uptake could cause decreases in RCT and cholesterol removal from the body, stemming from HDL dysfunction, increasing cardiovascular disease risk.

One open question is which cell receptors can engage in HDL holoparticle uptake. SR-BI was the first identified and scientifically accepted HDL-C receptor and exerts significant influence over HDL-C CE uptake levels [61]. However, SR-BI has not definitively been shown to engage in HDL holoparticle uptake. A study by Sun *et al.*, conducted in hepatocytes and fibroblasts, reported that cellular internalization of HDL from the blood via SR-BI results in the formation of two distinct pools of HDL. The first, much larger pool, is rapidly re-secreted into the blood with a half-life of approximately 10 min, whereas the second, smaller pool (approx. 4–7%) is endocytosed for holoparticle uptake via a lysosomal degradation pathway (figure 2) similar to the one taken by LDL after internalization by LDLR [62]. These results suggest that if SR-BI contributes to HDL holoparticle uptake, it is unlikely to do so to a significant extent. Experiments conducted by Pagler *et al.* in cell culture suggest HDL particles internalized by SR-BI are re-secreted rather than degraded, with HDL particles remaining attached to SR-BI as the receptor is internalized and being released when the receptor returns to the cell surface [63]. Such re-secretion does not account for holoparticle clearance of HDL by the liver. These cell culture experiments indicating that SR-BI does not play a key role in HDL holoparticle uptake are consistent with a report that in mice without functional SR-BI, no substantial change in HDL holoparticle uptake is observed [64]. Thus, even if SR-BI is capable of HDL holoparticle uptake, it is unlikely that it significantly contributes to this process.

Another member of the scavenger receptor B class of proteins, cluster of differentiation 36 (CD36), has more convincingly been shown to be capable of HDL holoparticle uptake. In a study conducted by Brundert *et al.*, HDL holoparticle uptake within the liver decreased by approximately 29% in mice lacking functional CD36 [56]. However, this leaves >70% of HDL holoparticle uptake by the liver unaccounted for. CD36 and SR-BI both function in HDL-C selective CE uptake in the liver, and their extracellular loops have high sequence homology [65]. Therefore, we might expect some redundancy in their roles in HDL-C uptake, similar to the one between LDLR and SR-BI in LDL-C uptake [18]. Surprisingly, in the study by Brundert *et al.*, SR-BI expression was unchanged in mice without functional CD36 [56]. Since SR-BI and CD36 are associated with different signalling pathways, increasing expression of one receptor without impacting the other could result in significant downstream health effects [12,66,67].

Of course, all findings from studies performed in mice and cultured mouse hepatocytes must be analysed critically. Sequence homology for both CD36 and SR-BI between humans and rodents is very high [66,68–71]. However, mice and humans have important differences that could limit the applicability of findings in mouse models to humans. For example, mice lack CETP, which transfers CEs between HDL and LDL [18,30,72]. In humans, transfer of CEs from HDL to LDL can reduce HDL’s effectiveness at removing cholesterol from the blood via RCT [20]. This, combined with the fact that the dominant form of cholesterol in mice is HDL, not LDL, is a significant limitation in our ability to accurately model the distinct impacts of each type of cholesterol [73,74]. Thus, validating findings in human cell lines and primary cells is essential for drawing more definitive conclusions about the molecular pathways involved in HDL holoparticle uptake.

Although the experimental findings discussed leave many unanswered questions, they also point towards possible therapeutic applications aimed at modulating HDL holoparticle uptake. Targeting molecules such as CD36 to reduce HDL holoparticle uptake could allow HDL particles to linger in the blood, where they could theoretically contribute to RCT and plaque clearance for longer. Experimental work supports the idea that CD36 loss of function confers atheroprotection, though the primary focus thus far has been on LDL-C-based mechanisms [67,75]. Oxidized LDL-C (oxLDL) promotes the formation of atherosclerotic plaques by preventing macrophages from migrating; when the macrophages become trapped in plaques, they propagate plaque growth [67]. *In vitro* and *in vivo*, oxLDL cannot prevent macrophages from migrating without functioning CD36 [67,75]. In addition, mice without functional CD36/ApoE cannot efficiently transport oxLDL into macrophages, reducing the formation of foam cells, a key component of atherosclerotic plaques [11,76,77]. Thus, mice without functional CD36/ApoE exhibit significant atheroprotection in various models of atherosclerosis [67], which reflects the effect of reduced CD36 levels on HDL holoparticle uptake.

Experimental evidence supports the feasibility of targeting CD36 to reduce the rate of HDL holoparticle uptake. As described, Brundert *et al.* reported that in mice, loss of CD36 function did not impact SR-BI expression in the liver [56]. This suggests it might be possible to decrease CD36 levels while simultaneously upregulating SR-BI expression to prevent removal of HDL-C selective CE uptake, which is necessary to remove cholesterol from the blood. This theory could be tested in mice in a model downregulating CD36 in the liver but would need to be validated in human cell lines and does not acknowledge the impact of macrophage CD36 and its contribution to atherosclerotic plaques [67,75]. If reducing CD36 levels to dampen HDL holoparticle uptake rates and boost atheroprotection works in mice, there is reason to think it could translate to the clinic. A variety of CD36-targeting drugs are currently being investigated for clinical use in the United States, and one, empagliflozin, has received tentative approval by the US Food and Drug Administration for a number of indications, including to treat type 2 diabetes and prevent deaths from heart failure [78–82]. Despite the profound impact of holoparticle uptake on the clearance of HDL-C particles from circulation, knowledge of the rate of uptake, how uptake occurs and the mechanisms by which it occurs are extremely limited. Further investigation into this function is likely to lead to treatments that can increase HDL-C efficiency and lifespan in the bloodstream, allowing it to continue removing excess cholesterol and improving health.

## 5. Mechanisms of high-density lipoprotein cholesterol selective cholesteryl ester uptake by hepatocytes

HDL-C selective CE uptake in the liver is the body’s main method of removing excess cholesterol, with disruptions in the expression of receptors and transporters within the selective uptake pathway augmenting cardiovascular disease risk [83,84]. Excess cholesterol in the bloodstream forms plaques along the blood vessel walls, causing decreased blood flow, cardiovascular disease and inflammation. The primary receptor that conducts HDL-C selective CE uptake is SR-BI. Though other receptors can conduct selective CE uptake, it is not their primary function, and they do not contribute to selective CE uptake as significantly as SR-BI [13–15,17,56]. Here, we review what is known about SR-BI’s role in HDL-C selective CE uptake and discuss how deeper knowledge of this process could translate into clinical gains.

**Figure 3.**
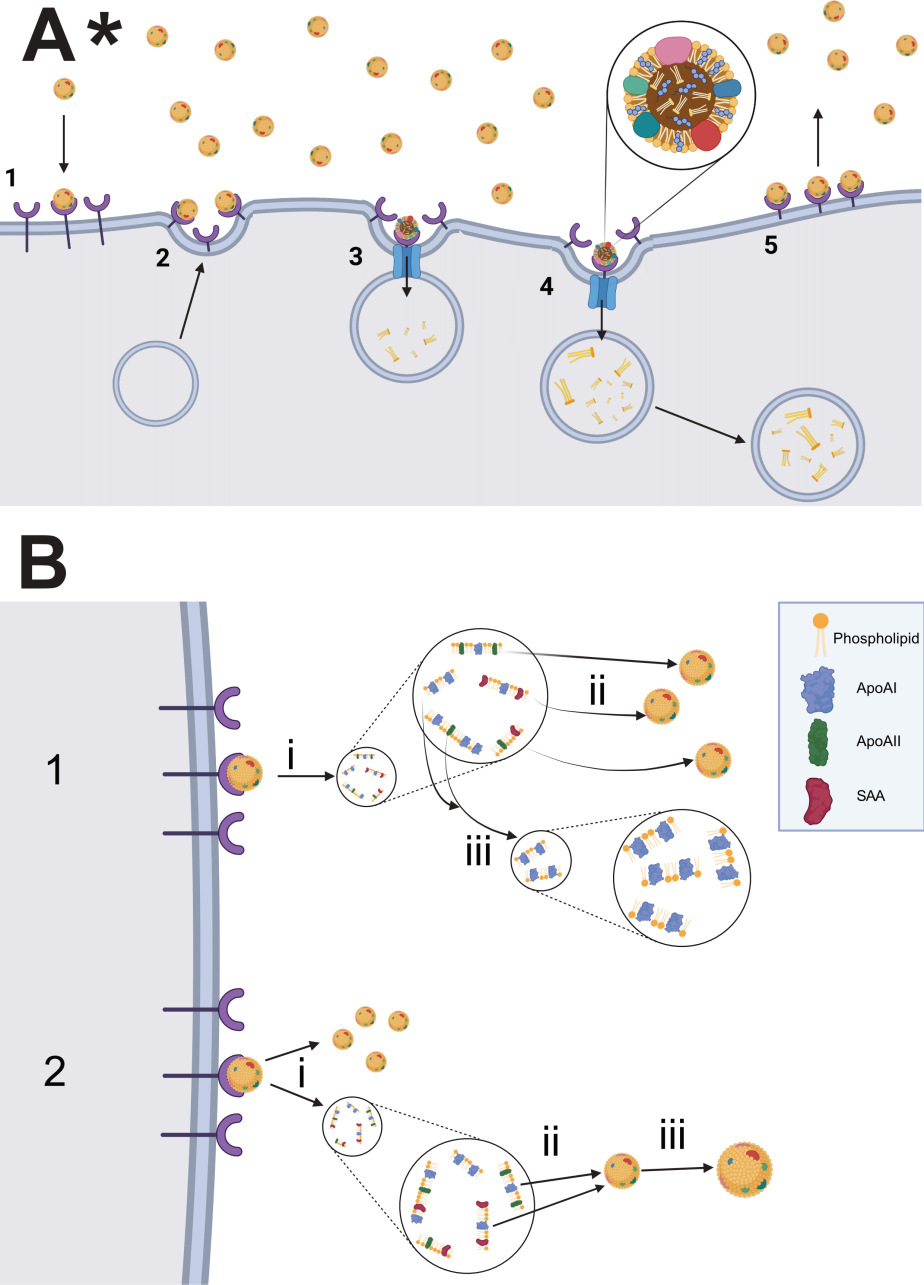
HDL-C cell surface selective uptake versus receptor fragmentation. (A*) (1 and 2) Plasma HDL-C particles bind to a transmembrane receptor. (3*) A transmembrane channel forms within the receptor, allowing the HDL-C particle’s cholesteryl esters to diffuse into the cell. (4*) Cholesteryl esters are released back into the cell and the transmembrane channel is closed. (5) HDL-C is released back into the plasma by the transmembrane receptor. (B) (1(i)). Fragmentation of HDL-C particle into various apolipoprotein and phospholipid fragments. (1(ii)) Re-integration of ApoAII- and SAA-containing particles into plasma HDL-C particles. (1(iii)) Further breakdown of ApoAI-containing fragments into smaller fragments. (2(i)) Breakdown of HDL-C particle into smaller HDL-C particle(s) and apolipoprotein and phospholipid fragments. (2(ii),(iii)) Re-integration of SAA- and ApoAII-containing fragments into plasma HDL-C, causing the particle to grow. *The opening and closing of the transmembrane channel remain a hypothesis. The way in which the channel forms continues to be researched.

Understanding the mechanism of selective CE uptake via SR-BI is crucial for the potential manipulation of this activity, which could increase cholesterol clearance and alleviate cardiovascular stress. As mentioned previously, the primary focus of HDL-C-based clinical targets has been upstream of the uptake process, focusing on increasing the levels of HDL-C and its components. Although we lack a complete SR-BI protein structure, evidence from studies of SR-BI and other proteins from the same class suggest that selective CE uptake occurs via a hydrophobic channel through SR-BI’s anti-parallel β-barrel core (figure 3A) [70,85–87]. Furthermore, SR-BI proteins bind to one another via disulfide bonds [66,88,89] and form multimers of up to 20 proteins within the cell membrane of hepatocytes [90–92]. Although this multimerization is not initiated by the presence of HDL-C [93], in human cell line experiments the size of SR-BI multimers increases with increasing concentrations of the experimental HDL-C analogue [90]. Furthermore, the concentrations tested fall below physiological averages, indicating that the size reported is likely an underestimation [90]. This corresponds with the positive association between HDL-C particle size and SR-BI binding strength [94], which could stem from HDL-C binding to multiple extracellular loops of SR-BI at once within the multimer, or because the bond between larger HDL-C particles and SR-BI may be stabilized by the attraction of the HDL-C to surrounding SR-BI proteins within the multimer. Combined, this suggests that the binding of SR-BI molecules to one another may facilitate the creation of a larger, dimer or multi-molecule hydrophobic channel that contributes to the increased CE transport and heightened binding strength of larger HDL molecules.

The presence of these SR-BI multimers complicates reports that HDL-C holoparticle uptake by SR-BI, however low a rate, occurs. If SR-BI multimerization is responsible for the creation or stabilization of the hydrophobic HDL-C CE transport tunnel, then the internalization and subsequent cycling back to the membrane of this large SR-BI block is extremely unlikely, due to its size and the major disruption it would cause to the cell membrane. In the experiments performed by Marques *et al.*, minimal internalization of SR-BI into hepatocyte cell lines occurred, and the internalization that did occur took place over upwards of 12 h, significantly longer than would be expected if internalization of the receptor was required for any meaningful level of HDL-C uptake [90]. Multiple groups have proposed that the detergent method utilized in earlier studies would have been unlikely to disturb the large, more stable multimers [90,95]. Therefore, it is likely that SR-BI monomers, as shown by the authors [90], and possibly even dimers or tetramers are maintaining reversible interactions with these multimers, connecting and disconnecting from the multimers at a rapid rate. This process was causing the previously observed endocytosis of receptor and particle, because the detergent was able to impact the weaker interactions of the single SR-BI proteins and/or smaller multimers and allow for their internalization [95]. Marques *et al.* also showed that mutations in SR-BI’s C-terminal leucine zipper, a previously proposed oligomerization motif, increased endocytosis rates, reducing HDL-C binding and increasing internalization of the apolipoprotein [71,88,96]. This is consistent with the idea that disturbing the capacity of SR-BI to multimerize results in the previously observed endocytosis of SR-BI.

Understanding how HDL-C selective CE uptake operates within the liver, both at the individual protein level and the interactions between multiple proteins, will likely lead researchers towards strategies to promote selective CE uptake and decrease the amount of cholesterol in the blood. While speculations have been made as to the function of these SR-BI multimers and the form of the transmembrane portion of SR-BI, these ideas need to be experimentally verified before significant progress can be made towards improving upon their current activity. As discussed previously, the increase in HDL levels alone has not shown significant benefits, likely because regardless of the level of HDL, selective CE uptake is still occurring at the same rate. Delving deeper into the mechanisms behind selective CE uptake via further investigation of the function of these SR-BI multimers and genetic variations in HDL receptors is the next logical step for moving excess cholesterol past the choke point of selective uptake and out of the bloodstream, where it leads to cardiovascular disease and atherosclerosis.

## 6. Plasma cycling of high-density lipoprotein cholesterol and its retention in circulation

Thus far, we have focused on longstanding questions about how HDL-C is taken up by liver cells to dispose of excess cholesterol. Equally important, however, is the lifespan of HDL particles and its correlation with HDL function. Cholesterol efflux capacity (CEC), or the ability of an HDL particle to remove cholesterol from peripheral cells, is likely impacted by the lifespan of the particle, as well as the previously mentioned CE uptake capacity of its receptors. This capacity is independent of overall HDL levels, meaning that a high number of HDL particles with a low CEC will not be as functional as a low number of HDLs with a higher CEC. The CEC of HDL particles has been shown to be tied to the apolipoprotein composition of the particles [97,98], as well as the activity of HDL associated enzymes such as paraoxonase [99–102] and hepatic lipase [102,103]. The duration of time for which an individual HDL particle remains, on average, in the bloodstream is unclear, and the pace at which these particles are cycled is likely to impact their function and the level of successful blood cholesterol clearance.

The dysfunction of HDL has been documented in patients with atherosclerosis [99], where the HDL particles were unable to inactivate or prevent the formation of oxidized phospholipids, key contributors to atherosclerotic plaques. Furthermore, data have shown that beyond not being able to fulfil its anti-atherosclerotic functions, dysfunctional HDL may even become pro-inflammatory and pro-atherosclerotic [104–108]. The longer an HDL particle remains in circulation, the more likely it is to be acted upon by forces that would cause its dysfunction. Therefore, while increasing HDL levels may be an appealing strategy for targeting atherosclerosis, doing so by preventing its clearance may only lead to further dysfunction. Instead, increasing HDL CEC by altering its apolipoprotein composition may be a compelling treatment target, a mechanism for which may already exist.

Evidence suggests that after HDL-C particles bind to SR-BI, they are fragmented. Using mouse models, De Beer *et al.* have shown that after HDL binds to SR-BI, small HDL fragments that contain both cholesterol and apolipoproteins are generated and circulate in the plasma [42,109,110]. It is not entirely clear whether every HDL particle that binds to SR-BI is broken down via this fragmentation process, whether the entire particle becomes fragmented, or whether only a portion is broken into fragments while the remainder of the particle simply shrinks in size (figure 3B). This fragmentation could represent a method for clearing the bloodstream of older HDL particles that have increased in size over time. Simultaneously, the re-integration of these HDL fragments into HDL particles in the plasma could be a mechanism for increasing HDL particle size to promote receptor binding [94,111] and increase storage capacity for CEs and triglycerides, boosting RCT rates.

The HDL fragments fall into three size classes. The apolipoprotein casing of each class contains a different amount of ApoAII and serum amyloid A (SAA), which are lipoproteins that contribute to the external casing of HDL particles and are capable of receptor binding. SAA has been shown to replace ApoAI as the dominant apolipoprotein component of HDL particles, influencing their receptor binding capabilities [54]. Whereas the fragments in the two largest classes can re-integrate into full size HDL particles within the plasma, fragments in the smallest class—which contain only ApoAI and lack ApoAII and SAA—cannot (figure 3B(i)) [42]. When sufficient HDL particles are not present in the plasma to accept HDL fragments, the larger size classes of fragments are broken down into the smallest class, which is then removed from the circulation [109]. When HDL fragments re-integrate into HDL particles, the particles’ binding strength to SR-BI increases because of their larger size [94,111]. This may in part be due to the shift of lipids normally stored in the core of the HDL particles to the surface of smaller HDL particles [112], which causes the apolipoprotein casing of the HDL particle to increase in hydrophobicity [94,111], interfering with binding to HDL receptors [112].

Higher amounts of ApoAII in HDL fragments are positively correlated with re-integration into HDL-C particles circulating in the plasma [42]. This re-integration increases the ratio of ApoAII in proportion to ApoAI within HDL particles, decreasing their binding affinity to SR-BI, as ApoAII is ordinarily negatively associated with binding affinity [17]. This indicates that though the increase in the ApoAII fraction of the HDL particle containing re-integrated fragments reduces its binding affinity, when the particle does bind SR-BI, it will remain there for longer and efflux more cholesterol. The HDL fragments generated by SR-BI processing also display a higher binding affinity for SR-BI than HDL, possibly as a negative feedback mechanism to prevent the removal of too much HDL-C from plasma circulation at once [109]. Furthermore, the higher the level of ApoAII within an HDL particle, the lower the rate of CETP remodelling of the particle [113]. Increased ApoAII levels would increase the rate of HDL-mediated cholesterol efflux, as CEs would be transferred to LDL at a lower rate. The correlation between increase in ApoAII, increase in size and fragment interference in HDL SR-BI binding, therefore, could allow the now larger particles to collect more peripheral cholesterol before being drawn to bind to hepatocytes.

The current knowledge on HDL-C plasma cycling is predominantly derived from experiments conducted in either Chinese hamster ovary cells or mouse cells, or in mice, so their physiological relevance to HDL processing in humans may be somewhat limited. In mice, an extremely low level of the HDL fragments has been documented [110], indicating either minimal production of these fragments, an extremely rapid re-integration rate or both. Regardless of their detectable presence *in vivo*, compelling evidence has been presented that these fragments are physiologically relevant and serve some function in altering HDL particle size, apolipoprotein composition and bloodstream longevity, which impact their binding and CE efflux capabilities, along with to which HDL receptor they are most likely to bind. HDL particles are not a monolith in their composition, and the levels of the apolipoproteins that compose them have implications for their function and level of cholesterol efflux [15,54]. The change in ratio of apolipoproteins within HDL particles impacts to which receptor they are most likely to bind, along with binding strength/frequency [15], which has immense downstream effects on inflammation, immune and endothelial cell function and more [12]. More research is needed on the impact of apolipoprotein composition, potentially leading to ways to affect receptor binding and downstream signalling. Furthermore, the increase in ApoAII for HDL particles containing re-integrated fragments impacts the rate at which CEs can be transferred by CETP to LDL, and modulating the production of the fragments could alter the ratio of HDL and LDL in therapeutically beneficial ways [113]. Better understanding of the function of these particles, their creation and their re-integration process is likely to lead to a more comprehensive understanding of how HDL levels and their efflux rates are maintained, opening avenues for the manipulation of these processes to alleviate cardiovascular diseases.

## 7. Conclusion: all hope is not lost for high-density lipoprotein cholesterol

HDL remains the far less understood or clinically successful sibling of LDL. To date, the vast majority of clinical trials showed no reduction of cardiovascular disease risk following the modulation of HDL levels. However, HDL plays a critical role in RCT and removing excess cholesterol from the body, a process key for preventing atherosclerosis. Thus, it is far too soon to abandon HDL metabolism as a potential source of novel therapeutic targets. Untangling the molecular pathways that underlie HDL-C uptake and cycling in the bloodstream may reveal promising new candidate agents. Key findings on HDL-C uptake and plasma cycling from mouse experiments should be verified using human cell lines to account for the discrepancies between mouse and human lipoprotein fractions. More research into genetic variants that are associated with cardiovascular disease may also prove helpful in identifying druggable targets [114]. Considering the complexity of HDL receptors and signalling pathways within lipoprotein metabolism, it is possible that truly effective therapies targeting atherosclerosis will need to be tailored to the individual based on their metabolic dysfunction. Alternatively, multiple agents targeting different aspects of lipoprotein metabolism may need to be combined to yield efficient results. In addition to combining treatments that target multiple points in HDL metabolism, combining treatments that increase RCT rates via HDL-based mechanisms and treatments that decrease LDL levels, such as statins, may improve outcomes for the many people currently suffering from cardiovascular disease.

## Data Availability

This article has no additional data.
